# Influence of Biochar and Bio-Oil Loading on the Properties of Epoxy Resin Composites

**DOI:** 10.3390/polym15081895

**Published:** 2023-04-15

**Authors:** Pamela Hidalgo, Luis Salgado, Nayadeth Ibacache, Renato Hunter

**Affiliations:** 1 Department of Industrial Processes, Faculty of Engineering, Universidad Católica de Temuco, Rudecindo Ortega 02950, Temuco 4780000, Chile; 2 Department of Mechanical Engineering, Universidad de La Frontera, Casilla 54-D, Temuco 4811230, Chile

**Keywords:** biochar, bio-oil, epoxy resin, pyrolysis, substitution, wheat straw, hazelnut

## Abstract

In this study, we evaluated the use of bio-oil and biochar on epoxy resin. Bio-oil and biochar were obtained from the pyrolysis of wheat straw and hazelnut hull biomass. A range of bio-oil and biochar proportions on the epoxy resin properties and the effect of their substitution were investigated. TGA curves showed improved thermal stability for degradation temperature at the 5% (T5%), 10% (T10%), and 50% (T50%) weight losses on bioepoxy blends with the incorporation of bio-oil and biochar with respect to neat resin. However, decreases in the maximum mass loss rate temperature (T_max_) and the onset of thermal degradation (T_onset_) were obtained. Raman characterization showed that the degree of reticulation with the addition of bio-oil and biochar does not significantly affect chemical curing. The mechanical properties were improved when bio-oil and biochar were incorporated into the epoxy resin. All bio-based epoxy blends showed a large increase in Young’s modulus and tensile strength with respect to neat resin. Young’s modulus was approximately 1955.90 to 3982.05 MPa, and the tensile strength was between 8.73 and 13.58 MPa for bio-based blends of wheat straw. Instead, in bio-based blends of hazelnut hulls, Young´s modulus was 3060.02 to 3957.84 MPa, and tensile strength was 4.11 to 18.11 Mpa.

## 1. Introduction

In recent years, many efforts have been devoted to utilizing lignocellulosic biomass as a valuable chemical resource for synthesizing polymers. Different methods have been developed to obtain a wide range of products from biomass, such as chemical and thermochemical processes [1]. Thermochemical processes mainly used for converting biomass into chemicals are combustion, pyrolysis, gasification, and liquefaction [2,3]. For example, some researchers have attempted to substitute liquefied wood with epoxy resin. Liquefied wood has high reactivity because of the large amount of phenolic OH groups and alcoholic OH groups [1,4,5,6]. Other studies reported the use of wood pyrolysis bio-oil [7,8].

In general, wood pyrolysis bio-oil is composed of water, acids, alcohols, aldehydes, esters, ketones, phenols, guaiacols, syringols, furans, lignin-derived phenols, and extractible terpenes with multifunctional groups [9,10,11,12]. Wood pyrolysis bio-oil is use since it contains phenols, aromatic hydrocarbons, and neutral components that can be substituted in the epoxy resin [13,14]. The availability of bio-oil -OH groups to epoxy resin is important in the cross-linking process, which is attributable to the reaction between the epoxide and hydroxyl (-OH) of bio-oil, which is an important factor that influences the bonding ability of the modified resin [8].

Moreover, epoxy resins are very versatile in nature. They are one of the most important classes of thermosetting polymers and are widely used as matrices for fiber-reinforced composite materials and as structural adhesives [15]. They are amorphous, highly cross-linked polymers, and this structure results in these materials possessing various desirable properties, such as greater tensile strength and modulus, uncomplicated processing, fine thermal and chemical resistance, and dimensional stability [16]. However, higher costs and brittleness limit its application in industry [17]. To improve its properties, fillers and additives have been incorporated, such as silica nanoparticles, montmorillonite, granite powder, carbon nanotubes, and wood dust [18,19,20]. It has been proven that the effect of wood dust on mechanical properties increases [15], and carbon nanotube/epoxy nanocomposite reinforcement improves thermal and mechanical properties [19,20]. Moreover, the effect of modifiers such as resorcinol phenyl phosphate on mineral fillers such as diorite on the physicochemical and deformation strength properties of epoxy-based composites has been studied [18].

At the same time, in the past few decades, research and engineering interest has been focused on the use of lignocellulosic biomass for obtaining green composites due to its recyclability and biodegradability, such as the use of wood pyrolysis bio-oils and biochar on the properties of epoxy composites.

Wood pyrolysis bio-oils (or liquid fraction) are obtained from biomass pyrolysis under anoxic conditions (non-oxidizing atmospheres) at high temperatures (~500 °C). Pyrolysis is one process of thermal decomposition of the biomass organic matrix resulting in the conversion of biomass into energy and chemical products, consisting of bio-oil, biochar, and non-condensable gas products (pyrolytic gas) [2]. Depending on the heating rate and residence time, aimed at maximizing either the bio-oil or biochar yields, biomass pyrolysis can be classified into three main types: slow or conventional, fast, and flash pyrolysis. [2,21,22,23,24].

Biochar has been used traditionally as a soil amendment due to its high nutrient contents (macro- and micro-nutrients). The concentration of respective nutrient elements varies over a wide range, depending primarily on the biomass type and pyrolysis conditions [21,25,26]. Recently, it has been used in materials applications, such as carbon fillers, because of its ability to improve the mechanical, electrical, and thermal properties of the final product [27,28,29]. With respect to bio-oil, studies have focused on blending it with epoxy resin. The results from these findings have confirmed the comparative lap-shear strength of bio-oil-based epoxy resins to commercial-grade epoxy resin and higher resistance to moisture [7].

This manuscript studies the use of pyrolytic products in epoxy resin composites. The effect of different biochar and bio-oil loadings on the epoxy composite was studied. Bio-oil and biochar were blended into the resin to form a cross-linked copolymer network structure, where the degree of reticulation, thermal stability, and mechanical performance were analyzed. This work provides an alternative for biomass revaluation of wheat straw and hazelnut hulls through pyrolysis to develop a novel epoxy resin cross-linked with bio-oil and biochar, thus analyzing the effect of pyrolytic products on the properties of the epoxy composite.

## 2. Materials and Methods

### 2.1. Methodology

Wheat straw and hazelnut hulls, and residual biomass generated in southern Chile, were used for the production of bio-oil and biochar. The epoxy resin, bisphenol A diglycidyl ether (DGEBA, molecular weight: 340.41 g/mol, CAS N° 1675-54-3) and tetraethylene entaamine (TEPA, molecular weight: 189.3 g/mol CAS N° 112-57-2), used as curing agents, were purchased from Sigma–Aldrich (Santiago, Chile). All chemicals were used as received and were of reagent grade.

### 2.2. Experimental Setup and Procedure

#### 2.2.1. Pyrolysis of Biomass

The biomass of wheat straw and hazelnut hulls was converted into biochar and bio-oil using a slow pyrolysis process operated under atmospheric pressure, with heating controlled by a data acquisition instrument. The pyrolysis of the samples was carried out at a temperature of 600 °C, with a residence time of the biomass in the pyrolyzer of approximately 3 h to ensure complete conversion. The reactor was continuously injected with nitrogen (1000 mL min^−1^). Then, the biochar was separated, milled, and fractioned using a sieve N°200 (U.S.A. Standard testing sieve, size < 75 µm). The bio-oil was collected and filtered to remove char particles using #1 Whatman paper. The characterization of pyrolysis products is shown in Table 1 and Table 2.

Compounds in bio-oil shown Table 2 were analyzed on a gas chromatograph (GC–MS-QP 2010 PLUS, Shimadzu, Kyoto, Japan), and it was configured under the following conditions: an HP5-MS fused silica capillary column (30 m × 0.25 mm × 0.25 mm); an oven temperature starting at 308 °K, rising to 453 °K at 5 °K min^−1^, and then up to 573 °K at 20 °K min^−1^; an injector temperature of 523 °K; helium (99.999%) used as a carrier gas at pressure mode control (10 kPa) and a flow rate of 0.56 cm^3^ min^−1^; a transfer line and ion source at 523 °K; electron energy at 70 eV. A quadrupole mass detector was operated in electron impact ionization mode. Data were obtained using the software GCMS solution (v2.53) and mass spectra databases (NIST08 and NIST08s). The samples were prepared by diluting 20 mg of bio-oil in 1 mL of acetone as the solvent, and fluoranthene was used as an internal standard.

#### 2.2.2. Preparation of Bio-Based Epoxy Blends

The epoxy resin was blended with bio-oil at weight proportions of 5% and 10%. Acetone pretreatment of the samples (bio-oil/DGEBA) was carried out according to Liu et al. (2017). The pretreated mixtures were then blended with biochar at weight proportions of 5% and 10% (as shown in Table 2) and then used in the preparation of bio-based epoxy blends.

Bio-based epoxy blends were prepared using TEPA as a curing agent. The bio-oil/DGEBA/biochar mixture and curing agent were homogeneously mixed in 1:0.1 weight proportions and then stirred for 10 min until a complete dispersion of biochar was reached. The dispersion of biochar is challenging due to the dense nature of the epoxy resin because increasing the weight fraction of biochar in epoxy increases the viscosity of epoxy [31]. Subsequently, blends were degassed in a vacuum desiccator attached to a vacuum pump to remove the entrapped air, poured into a silicone mold, cured at 50 °C for 8 h, postcured at 65 °C for 12 h in an oven (Memmert UF-260 universal), and stored at room temperature.

### 2.3. Analytical Techniques

#### 2.3.1. Thermogravimetric Analysis (TGA)

The thermal degradation of the bio-based epoxy blends was carried out using a thermogravimetric analyzer (DTG-60H, Shimadzu). Twenty milligrams of the sample was heated from 20 to 800 °C at a 10 °C/min heating rate under a nitrogen atmosphere. The weight loss of the sample was recorded as a function of the temperature.

In this way, the onset degradation temperature (T_onset_), the end degradation temperature (T_endset_), the temperature associated with a mass loss of 5%, 10%, and 50% (T5%, T10%, and T50%), the maximum mass loss rate temperature (T_max_), and the residual mass at 800 °C (Rm800) were determined.

#### 2.3.2. Raman Spectroscopy

All the samples were prepared according to the previous procedure for bio-based epoxy blends, using TEPA as a curing agent. The Raman spectra were registered using the Unchained Labs Raman spectrometer (model Hound, equipped with the 785 nm laser line). The samples were manually placed, and spectra were recorded from 300 to 3300 cm^−1^ with 1.5 cm^−1^ resolution. The laser spot was focused onto the sample by a 50× magnification objective lens of an Olympus microscope. The degree of reticulation (α) using the Raman spectra was calculated as:

$$\propto =100*\frac{I_{1275}^{O}-I_{1275}^{t}}{I_{1275\;}^{O}}$$

where $I_{1275\;}^{O}$ is the normalized intensity of the 1275 cm^−1^ peak at the beginning and $I_{1275}^{t}$ is the normalized intensity at time t. The peaks were normalized by dividing their intensities by that of the 1160 cm^−1^ peak corresponding to the phenyl ring [32].

#### 2.3.3. Mechanical Analysis

**Figure 1:**
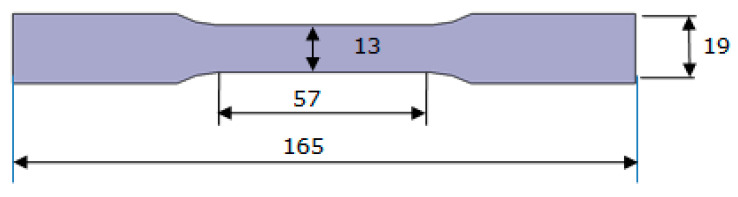
Geometric dimensions of dogbone specimens Type IV (ASTM D638).

Mechanical properties (tensile strength, tensile deformation, and modulus) were determined in a universal testing machine (model 3369, INSTRON, Norwood, MA, USA) and were evaluated according to ASTM D638. A type IV test specimen with a thickness of 4 mm was used (Figure 1). The samples were prepared using TEPA as a curing agent and were poured into a silicone mold according to the model for their preparation. The curing process was implemented in a Memmert UF-260 universal oven (Memmert, Schwabach, Germany) for 8 h at 50 °C and postcured at 65 °C for 12 h. Data acquisition and processing were performed using Instron Bluehill^®^ Lite (version 2.24) software. The tests were performed at room temperature, considering a constant displacement speed of 1 mm/min. An extensometer was used to measure specimen displacement.

## 3. Results and Discussion

### 3.1. Thermal Degradation Properties

**Figure 2:**
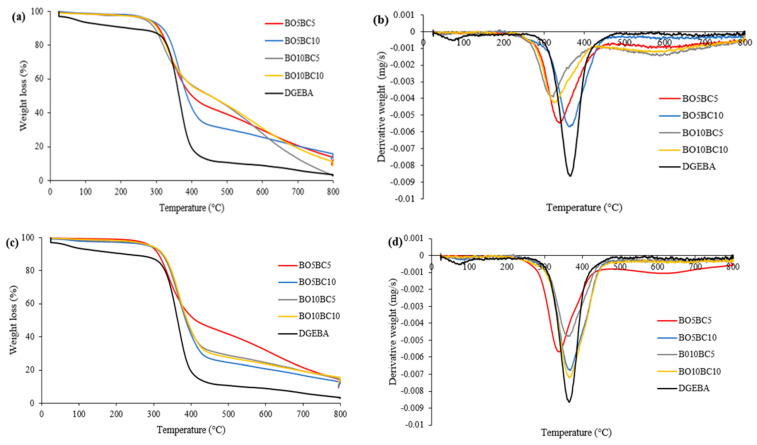
TGA (**a**) and derivative weight loss (DTGA) (**b**) curves for cured neat epoxy resin (DGEBA) and cured bio-based epoxy blends of wheat straw. TGA (**c**) and derivative weight loss (DTGA) (**d**) curves for cured neat epoxy resin (DGEBA) and cured bio-based epoxy blends of hazelnut hulls.

Figure 2 shows variations in the TGA and derivative TGA (DTGA) curves of neat epoxy resin and the bio-based epoxy blends. The temperatures corresponding to mass loss are presented in Table 3. The first step of the TGA curve is attributable to the dehydration of water, which occurs between room temperature and 120 °C [33]. The weight loss during the dehydration stage was approximately 7% for neat resin and 2% for bio-based epoxy blends of wheat straw. The neat epoxy resin showed T_onset_ at 320 °C and T_endset_ at 420 °C, with 3.04% residual mass. For blends of wheat straw, T_onset_ was between 295 and 320 °C, T_endset_ was approximately 420 °C, and the residual mass was between 2.76 and 15.05%, indicating that the addition of biochar and bio-oil produced a change in the thermal degradation. The T_endset_ values are practically unaffected by biochar and bio-oil addition, as opposed to T_onset_.

For blends of hazelnut hulls, T_onset_ was between 300 and 325 °C, T_endset_ was approximately 400 °C, and the residual mass was between 12.05 and 15.50%.

In addition, T5%, T10%, and T50% progressively increased with bio-oil and biochar incorporation with respect to neat resin. Higher T50% values with 10% bio-oil were observed independent of the biochar dosage of wheat straw, and T5% and T10% values slightly increased with increasing biochar dosage. This can be due to aromatic compounds of bio-oil, such as phenolic hydroxyls, that could react with the epoxy groups of resin, increasing the thermal stability [8], and the bio-oil of wheat straw has higher aromatic compounds than the bio-oil of hazelnut hull (such as phenol and aromatic hydrocarbons), as shown in Table 2. Bio-oil of the hazelnut hulls contained 35.47% aromatic compounds instead of 43.04% of the bio-oil of wheat straw that could be substituted in the epoxy resin.

From the DTGA curves, the maximum mass loss rate temperature (T_max_) reached was 365.72 °C in the neat resin, which is attributable to the degradation of the epoxy group. However, the addition of bio-oil and biochar produced a lower degradation resistance with respect to the neat epoxy resin, as observed in the derivative mass loss curves, diminishing the temperature associated with the maximum mass loss rate. The residual mass to 800 °C for neat epoxy resin was near 3%. However, in general, bio-oil and biochar incorporation led to an increase in Rm800.

### 3.2. Cross-Linking Analysis

**Figure 3:**
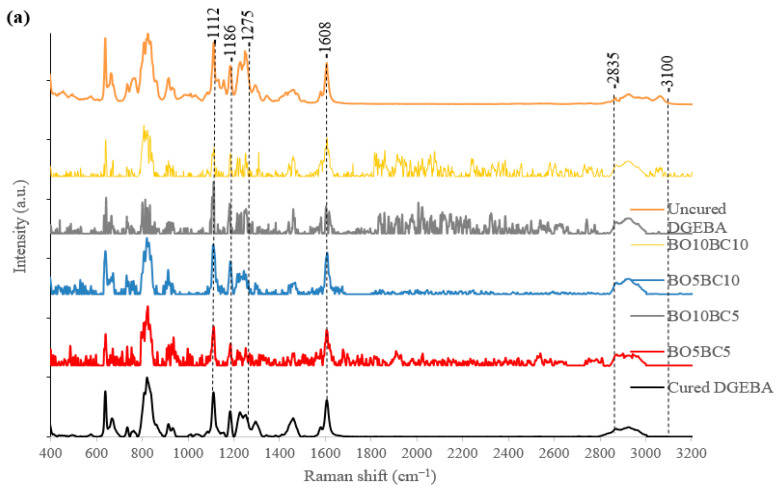

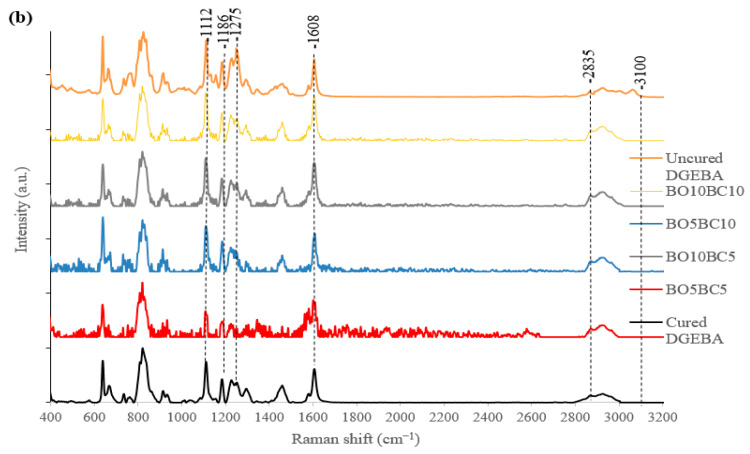
Raman spectra of (**a**) uncured neat epoxy resin (DGEBA), cured neat epoxy resin (DGEBA), and cured bio-based epoxy blends of wheat straw and (**b**) uncured neat epoxy resin (DGEBA), cured neat epoxy resin (DGEBA), and cured bio-based epoxy blends of hazelnut hulls.

Figure 3 shows the Raman spectra of uncured DGEBA, cured DGEBA, and cured bio-based epoxy blends. The Raman band corresponding to epoxide vibration is at 1275 cm^−1^, and the intensity of this peak is linearly dependent on the concentration of epoxide groups in the resin mixture [32,34]. During chemical curing, epoxide groups in epoxy resin react with hardeners, forming a highly cross-linked three-dimensional network, diminishing the peak intensity at 1275 cm^−1^ corresponding to the epoxide group, as observed in the Raman spectra of cured DGEBA and cured bio-based epoxy blends. This can be interpreted as the consumption of the free epoxide groups during the vitrification of the epoxy resin curing process [35]. In addition, a -CH_2_ stretching band at 2835 cm^−1^ associated with the formed link between the amine group of the hardener and the epoxy group of the resin has been observed in cured samples [36]. With respect to the Raman band of the -OH group present in the bio-oil, the region that appears, in accordance with the literature, is approximately 3000–3500 cm^−1^ [37,38,39,40,41]. However, this band is not observed in the samples. This confirmed the consumption of -OH groups upon reaction with DGEBA to generate a chemical bond [8]. Biochar infiltrates into resin through its pores, forming strong bonding, producing an increase in mechanical properties and enhancing cross-linking [28]. Other Raman peaks at 1112 cm^−1^, 1186 cm^−1^, and 1608 cm^−1^ are assigned to resin backbone vibrations that remain unchanged throughout the curing reaction. Raman peaks at 1112 cm^−1^ and 1186 cm^−1^ correspond to the C–C stretch. The Raman peak at 1608 cm^−1^ is assigned to the stretching of the phenyl ring [42]

The degree of crosslinking in the bio-based epoxy blend is approximately 70%, comparable to 72.161% of cured neat epoxy resin. This could indicate that bio-oil containing mainly phenols and aromatic hydrocarbons was substituted into the epoxy resin. Thus, incorporating bio-oil and biochar slightly modifies the epoxy group’s degree of reticulation (see Table 4). The addition of bio-oil and biochar does not significantly affect the mobility of molecules in chemical curing. The mobility of molecules diminishes during the curing process, thus increasing the degree of cross-linking [43,44].

For the bio-based epoxy blend, when increasing the biochar and bio-oil content obtained from wheat straw from 5% to 10%, a slight increase in the degree of crosslinking, approximately 70 to 72%, respectively, was observed. Moreover, in samples with 5% and 10% biochar, and with 5% bio-oil obtained from hazelnut hull, a mild decrease in the degree of crosslinking was observed. This could be due to the higher particle size of biochar. Instead, for a bio-oil content of 10%, no significant change in the degree of crosslinking was observed. This is indicative that aromatic compounds of bio-oil react with the epoxy group.

### 3.3. Mechanical Properties

**Figure 4:**
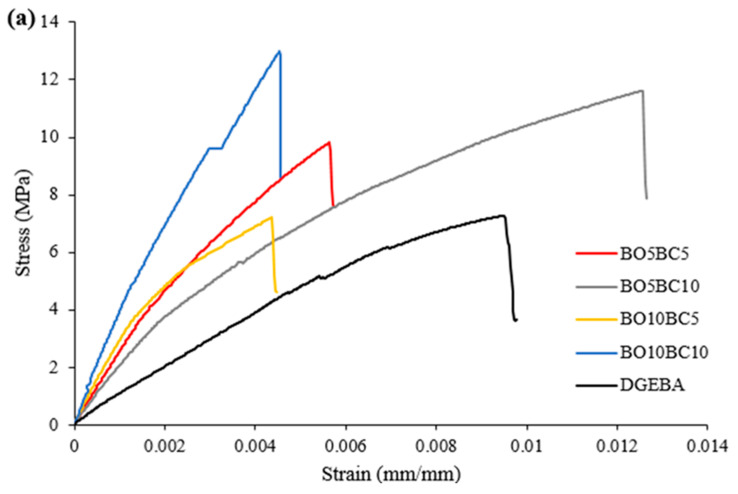

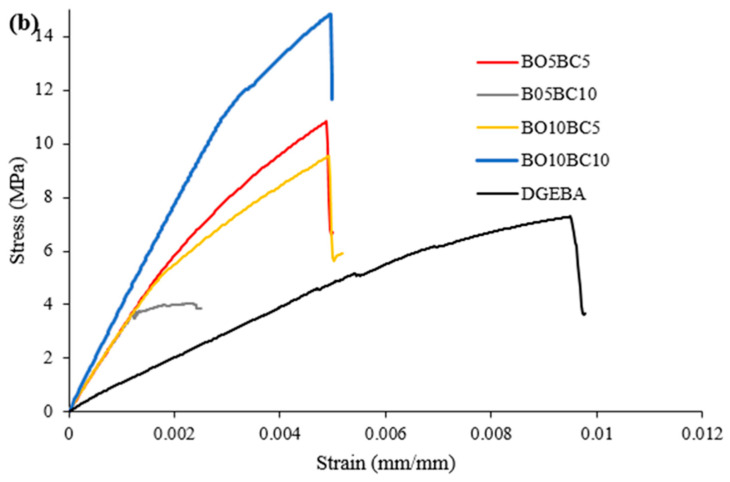
Representative stress–strain curves of (**a**) cured neat epoxy resin (DGEBA) and cured bio-based epoxy blends of wheat straw and (**b**) cured neat epoxy resin (DGEBA) and cured bio-based epoxy blends of hazelnut hulls.

Figure 4 shows the stress–strain curves of the neat epoxy resin and bio-based epoxy blends of wheat straw and hazelnut hulls. Table 5 shows the mechanical properties of the samples tested. All bio-based epoxy blends with biochar and bio-oil show a high increase in Young’s modulus with respect to neat resin. Young´s modulus was approximately 1955.90–3982.05 MPa for bio-based blends of wheat straw and 3060.02–3957.84 MPa for hazelnut hull.

With respect to tensile strength, in general, an increase was observed with respect to neat resin. For epoxy blends, biochar-bio-oil from wheat straw was in the range of 8.73 and 13.58 MPa. Instead, tensile strength was 4.11 and 18.11 Mpa for blends with biochar and bio-oil from hazelnut hulls. However, a low tensile strength was observed when a 5:10 proportion of bio-oil:biochar (BO5BC10) was added to the epoxy resin of hazelnut hulls. This proportion produced a decrease in cross-linking of the sample (as shown in Table 4), which could be due to deviation from the optimal stoichiometric ratio, leading to a reduction in tensile strength. For optimum cross-linking to occur, without affecting its properties, the precise molar ratio of the epoxy to hydroxyl group blend is critical before curing, while ensuring the highest thermal and mechanical properties of the bio-modified polymer [13,45].

Moreover, an increase in tensile strength with the addition of biochar at 5 to 10% was observed, with a higher use of biochar from hazelnut hull as a filler. This increase could be due to biochar particle size. The filler particle size of biochar of hazelnut hulls is higher than that of wheat straw, as shown in Table 1. The filler particle size is another important factor that contributes to composite properties. It has been reported that the particle size of filler can greatly alter composite properties, and a decrease in mechanical properties has been observed with very small particle sizes. This decrease is because of greater interfacial adhesion between the matrix, and filler having a smaller particle size [46]. Moreover, a factor contributing to the decline in tensile strength is the aspect ratio of the filler [47]. A larger aspect ratio decreases the tensile modulus of the composite, and this increases with particle size [48,49].

## 4. Conclusions

Bio-oil and biochar were successfully incorporated into epoxy resin, increasing mechanical properties, such as tensile strength and Young’s modulus with respect to neat resin. The aromatic compounds of the bio-oil participated in the cross-linked network and did not significantly modify the degree of cross-linking with the increase in bio-oil. Moreover, biochar improves the mechanical properties with respect to neat resin, but this improvement is more significant using smaller filler particle sizes. A decreased thermal stability upon the addition of biochar and bio-oil compared to the thermal degradation of the neat polymer was observed. The onset of thermal degradation for neat resin was observed at 320 °C; however, with the addition of biochar and bio-oil, the temperature for degradation was between 295 °C and 325 °C, and within 2.95% and 15.50% biochar content. This study is industrially novel because the greatest improvement has occurred in mechanical properties with bio-oil and biochar incorporation into epoxy resin without the risk of diminishing thermal properties.

## Figures and Tables

**Table 1:** Properties of biomass and biochar pyrolyzed at 600 °C. Adapted with permission from Hidalgo et al (2019) [30].

	Fixed Carbon (%)	Volatile Matter (%)	Ash (%)	Moisture (%)	N (%)	C (%)	H (%)	O (%)	H/C Molar Ratio	O/C Molar Ratio	Mean Particle Size (μm)	Surface Area (m^2^/g)	Pore Volume (cm^3^/g)	Pore Diameter (Ȧ)
Feedstock														
Wheat straw	16.11	70.41	4.75	8.61	0.63	43.11	5.81	50.45	1.62	0.80				
Hazelnut hull	37.32	51.7	1.37	8.89	0.11	35.98	6.00	57.91	2.00	1.18				
Biochar pyrolyzed														
Wheat straw	61.89	17.44	15.61	4.84	0.36	66.27	2.21	31.16	0.40	0.18	11.7 ± 0.7 *	57.388	0.01	10.4
Hazelnut hull	88.27	5.86	4.65	1.21	4.36	89.82	1.70	4.12	0.23	0.002	20.9 ± 0.6 **	18.033	0.005	10.5

Accumulate size distribution of biochar particles: * 25%: 5.3 μm, 50%: 10.2 μm, 75%: 15.1 μm; ** 25%: 7.1 μm, 50%: 20.1 μm, 75%:25.5 μm.

**Table 2:** Composition of bio-oil.

	Relative Content (%)	
Composition	Wheat Straw	Hazelnut Hull
Aromatic compound:		
Aromatic hydrocarbon	6.67	7.1
Phenols	36.37	28.37
Aliphatic hydrocarbons	26.24	18
Ketone	10.22	9.22
Ether	-	20
Amine	5	-
Non identified	10.5	17.31

**Table 3:** Thermal degradation behaviors for cured neat epoxy resin (DGEBA) and bio-based epoxy blends.

Sample	T_onset_ (°C) ^a^	T_endset_ (°C) ^b^	T5% (°C) ^c^	T10% (°C) ^d^	T50% (°C) ^e^	T_max_ (°C) ^f^	Rm800 (%) ^g^
DGEBA	320	420	73.01	222.37	363.9	365.72	3.04
Wheat straw							
BO5BC5	305	420	278.17	307.78	401.31	338.31	12.35
BO5BC10	320	422	280.27	317.93	386.87	365.11	15.05
BO10BC5	300	425	269.13	295	453.46	323.33	2.76
BO10BC10	295	420	277.59	303.22	452.53	325.99	11.06
Hazelnut hull							
BO5BC5	300	400	291.44	312.52	412.83	338.19	12.83
BO5BC10	320	425	286.93	323.04	385.50	367.85	12.05
BO10BC5	325	420	290.55	321.94	387.33	361.36	13.54
BO10BC10	320	420	293.30	325.29	389.71	365.08	15.50

^a^ Initial degradation temperature (°C). ^b^ Final degradation temperature (°C). ^c^ 5% weight loss temperature (°C). ^d^ 10% weight loss temperature (°C). ^e^ 50% weight loss temperature (°C). ^f^ maximum mass loss rate temperature (°C). ^g^ Residue (wt%) at 800 °C.

**Table 4:** The degree of crosslinking for cured neat epoxy resin (DGEBA) and bio-based epoxy blends.

Sample	Degree of Crosslinking (α)
DGEBA	72.161
Wheat straw	
BO5BC5	70.014
BO5BC10	71.686
BO10BC5	70.965
BO10BC10	71.832
Hazelnut hull	
BO5BC5	72.879
BO5BC10	69.881
BO10BC5	71.739
BO10BC10	71.630

**Table 5:** Mechanical properties of cured neat epoxy resin (DGEBA) and bio-based epoxy blends.

Sample	Tensile Strength (MPa)	Max. Deformation (mm/mm)	Young’s Modulus (MPa)
DGEBA	7.52 ± 1.09	0.010	1093.69 ± 128.83
Wheat straw			
BO5BC5	10.96 ± 1.82	0.005	2519.38 ± 194.37
BO5BC10	13.58 ± 3.97	0.010	1955.90 ± 83.35
BO10BC5	8.73 ± 0.85	0.00	3091.31 ± 362.84
BO10BC10	13.02 ± 9.3	0.005	3982.05 ± 582.65
Hazelnut hull			
BO5BC5	14.04 ± 6.30	0.006	3060.02 ± 252.49
BO5BC10	4.11 ± 1.86	0.003	3138. ±19.55
BO10BC5	10.93 ± 2.49	0.005	3270.30 ± 1470.47
BO10BC10	18.11 ± 3.35	0.005	3957.84 ± 130.91

## Data Availability

The data presented in this study are available on request from the corresponding author.
